# General practitioners' decision making managing uncomplicated urinary tract infections in women: a qualitative study

**DOI:** 10.3399/BJGPO.2023.0224

**Published:** 2024-07-10

**Authors:** Peter K Kurotschka, Juliane Hemkeppler, David Gierszewski, Luca Ghirotto, Ildikó Gágyor

**Affiliations:** 1 Department of General Practice, University Hospital Wurzburg, Wurzburg, Germany; 2 Qualitative Research Unit, Azienda USL – IRCCS di Reggio Emilia, Reggio Emilia, Italy

**Keywords:** qualitative research, family medicine, prescribing, urinary tract infections, general practice

## Abstract

**Background:**

To be effective, interventions aimed at increasing the appropriateness of antibiotic use in primary care should consider the perspectives of prescribing physicians.

**Aim:**

To explore the decision making of general practitioners (GPs) when managing uncomplicated urinary tract infections (uUTIs) in women.

**Design & setting:**

A qualitative study using semi-structured interviews with 22 GPs in Bavaria and Baden-Württemberg (southern Germany).

**Method:**

Verbatim transcripts were analysed through inductive qualitative content analysis.

**Results:**

We generated the following three main themes: factors facilitating the decision making; factors complicating the decision making; and consultation modalities. According to participants, following evidence-based recommendations makes the prescription decision smoother. GPs' and patients’ prior experiences and beliefs guides decisions towards certain antibiotics, even if those experiences and beliefs contradict evidence-based recommendations. Patient expectations and demands also condition antibiotic prescribing, favouring it. Organisational constraints, such as time pressure, the day of the week (for example, before weekends), and a lower cost of antibiotics for patients than alternative treatments favour the decision to prescribe antibiotics. Diagnostic and prognostic uncertainty complicates decision making, as does scepticism towards evidence-based recommendations. Discordance within the patient–doctor relationship contributed to this complexity. Regarding consultation modalities, a more in-depth consultation and shared decision making were seen as helpful in this process.

**Conclusion:**

We identified different factors as intervening against or for a straightforward management decision when dealing with women with uUTIs. They reveal the complexity behind the GPs’ decision making. Providing GPs with easy-to-apply guidance while removing economic constraints to allocate sufficient consultation time, and supporting shared decision making may help GPs appropriately manage uUTIs in women.

## How this fits in

Urinary tract infections (UTIs) are common reasons for women to consult their general practitioner (GP). With causative bacteria becoming increasingly resistant to antimicrobials, understanding the GPs' decision making when managing UTIs in women is important to promote appropriate treatment approaches for this condition. This research highlights that managing UTIs in women results from a complex interplay among patient-, physician-, and healthcare system-related factors. It suggests that offering improved, easily applicable guidance, supporting shared decision making with patients, and providing financial incentives to allocate sufficient consultation time may assist GPs in navigating this complexity, ultimately enhancing clinical decision making.

## Introduction

Urinary tract infections (UTIs) are common reasons women consult a healthcare professional.^1–3^ Female patients with acute cystitis, that is, acute uncomplicated UTI (uUTI), are usually diagnosed and managed in primary care by GPs. As management guidelines recommend, its elective treatment predominantly consists of antibiotic therapy,^4,5^ although evidence suggests that many women may benefit only marginally from those drugs.^3,6^ Therefore, with antimicrobial resistance on the rise,^7^ primary care-directed antimicrobial stewardship (AMS) interventions to improve the management of uUTI in women are essential to reduce the use and misuse of antibiotics. Such efforts are directed either to foster guideline adherence in the choice of the right antibiotic, and the correct dose, at the right time,^8–10^ to find more accurate diagnostic pathways,^11,12^ or to use treatments other than immediate antibiotics, such as delayed prescribing strategies, herbal formulations, or painkillers.^6^

Antibiotic prescribing is a complex phenomenon influenced by context-specific factors, such as the sociocultural context, the healthcare system, or the nature of the disease.^13–18^ Qualitative research aims for a deeper understanding and interpretation of phenomena. It is best suited to explore the views, perspectives, and experiences of the healthcare professionals involved in the prescribing process. These insights can, in turn, crucially inform the design, conduct, and evaluation of AMS interventions, as suggested elsewhere.^19,20^ So far, most qualitative studies carried out in primary care have focused on clinicians' decision making in the management of respiratory tract infections.^13,17,21–25^ Despite the high prevalence of UTIs, there remains a lack of investigation into factors that influence the management decisions of GPs when dealing with women experiencing uUTI.^26–30^

Investigating what guides the GP’s agenda when decisions have to be made in this regard, would allow a more profound understanding of uUTI management. With the study reported here, we aimed to grasp GPs' perspectives on decision making when managing uUTIs in women.

## Method

We employed a qualitative study design using semi-structured interviews to answer the following research question: which factors influence the GP’s decision-making process when dealing with women suffering from an acute uUTI?

### Sampling and research setting

We opted for convenience sampling of GPs. In September 2019, we sent an invitation letter to participate in the study via post to 142 randomly selected GPs registered in Würzburg and the surrounding area (within the federal state of Bavaria) and in the northern part of Baden-Württemberg, Germany. We aimed to achieve a sample of 20–25 GPs and expected a response rate of 15–20% based on previous experience.^31^ Letters were enclosed in an official envelope with the university logo and contained an explanation of the study and data protection regulations. GPs were asked to communicate their willingness to be interviewed to the Department of General Practice of the University Hospital Würzburg (UKW) via telephone, fax, or email. In addition, the fieldwork coordinator (JH) invited three GPs from the northern region of the federal state of Bavaria to participate in the study. They knew them personally as being particularly interested in the topic and willing to participate in research projects.

Only physicians meeting the inclusion and exclusion criteria highlighted in Table 1 were invited to participate (further information on these criteria are provided in the Supplementary methods text).

**Table 1.** Inclusion and exclusion criteria

Inclusion criteria
Inclusion criteria Being an owner of, or employed in, a general practice: either a solo practice, a group practice, or a healthcare centreAND Being: a specialist in general practice or family medicine OR specialist in internal medicine OR without specialisation ('practical doctor')
Exclusion criteria GPs acting exclusively in the private insurance system GPs in training

### Data collection

We used a pre-planned topic guide for interviewing shown in the Supplementary Table S1. We defined specific foci on the uUTI management experience of GPs (diagnosis, treatment proposal, particular complex events, and expectations) without following any prior theoretical framework. We planned to use the first two interviews to pilot the topic guide; no adjustments were made, however, as they were not perceived as necessary by the interviewer. Data collection took place between October and December 2019. One researcher (JH, medical doctoral researcher in general practice and medical student at the time of data collection) conducted all semi-structured interviews face to face, in person, and in German, at the general practice (except for one, which was conducted at the GP's home). German was the native language of all participants and the interviewer. Interviews were paused if any interruption occurred. At the end of each interview, GPs were asked to provide sociodemographic information (age, practice size, rural or city location of their practice).

### Data analysis

Analyses were conducted concurrently to data collection and data collection was stopped after achieving a sample of 22 interviewed GPs, as the analysts felt that no new concepts emerged from the analyses (data saturation was achieved).^32^

All the interviews were audio-recorded and transcribed verbatim^33^ by one researcher (JH) soon after their conduct. Transcribed data were analysed employing the qualitative content analysis described by Kuckartz^34^ and using MAXQDA Analytics Pro 2018 by two researchers (JH, and DG, sociologist). They read all transcripts to familiarise themselves with the data and get an overall picture. Transcripts were fractured into manageable sections deductively following the interview topics. Then, the two analysts performed line-by-line coding of the first four interviews independently. Resulting codes of similar content were grouped into themes, thus defining two separate frameworks. After team discussions on the frameworks involving JH, DG, and IG (professor for general practice and practising GP), they were merged to create a shared codebook. This was applied to the remaining interviews. Once themes were generated from the whole dataset, codes and corresponding quotations were translated in English by JH and PK. Themes and sub-themes were then subsequently refined and renamed through team discussions involving JH, DG, IG, and two other authors naïve to the analyses (PK, academic GP, and LG, qualitative research methodologist), who acted as external audit and evaluators of the themes against the research question.

### Ethics and reporting standards

Written informed consent was obtained before the interview. Participants were informed of their right to withdraw from the study at any time. All interviews were de-identified by one researcher (JH) using MAXQDA Analytics Pro 2018. The study is reported following the Consolidated Criteria for Reporting Qualitative Research (COREQ) guidelines.^35^ Quotations from interviews are reported throughout the Results section. Additional quotations illustrating themes and sub-themes are available in the Supplementary Table S2.

## Results

A total of 22 GPs were interviewed, of whom 19 were recruited among the 22 GPs who responded to the initial postal invitation (response rate = 22/142 = 15.5%), and the remaining three were those personally contacted by JH. Sociodemographic characteristics are shown in Table 2. Notably, 73% of the GPs were based in rural or small-town practices.

**Table 2.** Participants' sociodemographic characteristics

Characteristic	Participants, *n* (%)	
Sex	Male	13 (59)
	Female	9 (41)
Age	<30 years	0 (0)
	30–50 years	12 (55)
	>50 years	10 (45)
Practice size	Solo practice	8 (36)
	Group practice or healthcare centre	14 (64)
Number of inhabitants of the town of the practice	<5000	9 (41)
	5000–20 000	7 (32)
	>20 000	6 (27)

We generated the following three main themes, each influencing the GPs’ management decisions when dealing with women with an acute uUTI (Table 3): factors facilitating the decision making; factors complicating the decision making; and consultation modalities.

**Table 3.** Themes and sub-themes

Theme	Sub-theme
Factors facilitating the decision making	Following evidence-based recommendations.GPs' prior experiences with antibiotics.Patients' prior experiences with antibiotics.Going along with patients' expectations and demands.Organisational constraints.
Factors complicating the decision making	Diagnostic and prognostic uncertainty.Scepticism towards evidence-based recommendations.Discordance within the patient–doctor relationship.
Consultation modalities	A more in-depth consultation.Shared decision making with patients.

### Factors facilitating the decision making

This category identifies situations that eased the GPs’ management decisions. Straightforward decisions were facilitated by some circumstances, identified as the following sub-themes: following evidence-based recommendations; GPs’ and patients’ prior experiences with antibiotics; going along with patients’ expectations and demands; and organisational constraints.

#### Following evidence-based recommendations

When deciding on a prescription, the participants reported using information from clinical guidelines and other official sources such as direct healthcare professional communications (Dear Doctor letters). Clinical guidelines were generally viewed as a respectable source of information, and their recommendations on first-line antibiotics were reported to be followed:

*'According to guideline recommendations. Fosfomycin. Or Pivmecillinam, for example. That’s definitely what I'm trying to do first*.' (B11)

Accordingly, if symptoms were not severe and no other risk factors for complications were present, GPs pursued a 'wait-and-see' approach while sending a urine sample for culture or prescribed alternative treatments:

*'The symptoms, the intensity of the symptoms, essentially decides it. You can then also offer the patient to send in a urine sample for culture*.' (B4)

*'If I have a patient* [...] *who has no major symptoms and no other risk factors, then I encourage them to drink a lot and ultimately maybe work with some herbal remedies to get the problem under control.'* (B16)

#### GPs’ prior experiences with antibiotics

Prior experiences with specific antibiotics appeared to shape beliefs about them that, in turn, were described as influencing the subsequent management decisions. For instance, the experience of vaginal mycoses after longer courses of antibiotics led to uUTIs being treated more frequently with a single-dose antibiotic:

*'And then we often give fosfomycin,* [...] *because the other preparations that are given for a long time often lead to fungal infections in the vagina.* […]*'* (B8)

The willingness of GPs to treat uUTIs with certain antibiotics depended on the perceived effectiveness defined by previous experiences:

'[…] *The single-shot, fosfomycin, I don't like that. Because you can't get rid of the total amount of bacteria with one dose*.' (B14)

Past experiences also concurred to define beliefs about local resistance levels influencing the choice of a specific antibiotic, sometimes despite guideline recommendations:

*'I'm still a big fan of fluoroquinolones* [...]*. I don’t feel that there is a high resistance rate here*.' (B16)

#### Patients' prior experiences with antibiotics

Participants perceived that patients reporting their experiences with medications for previous uUTI episodes influenced their prescribing attitude because *'they do tell you which pills have helped, which have not*' (B10).

One participant stated that patients’ prior experiences were even more important than guideline recommendations:

*'Well, if they've had it before and it helped them, then I usually write it down again* [...]*, why should I prescribe something else now? For me, the guideline is rather secondary* [in such cases].' (B9)

#### Going along with patients’ expectations and demands

Participant decision making was also influenced by patients' expectations and demands of antibiotics (which participants reported going along with).

From GPs’ perspectives, patients sometimes expect antibiotics to *'have eliminated the symptoms by tomorrow'* (B3). Especially when symptoms were severe, some GPs felt encouraged to prescribe an antibiotic immediately, as they perceived the patient expecting it:

'[…] *if they already have massive complaints, they want something that takes effect as soon as possible and then they get that on the first presentation, of course*.' (B4)

Regarding patient demands, GPs reported that sometimes patients explicitly requested the prescription of a specific antibiotic. Some of them tended to yield to patients' wishes without questioning:

*'If someone comes and wants Amoxiclav, then they get it*.' (B1)

#### Organisational constraints

Finally, considering organisational constraints facilitated a decision. Being under time pressure during a consultation appeared to favour the prescription of an antibiotic:

*'If you're under pressure, you don't have time, and there are ten people are sitting outside, you don't start discussions. You say okay, then you'll get an antibiotic, and goodbye if you're happy with it*.' (B11)

Moreover, the day of the week that the woman presents with uUTI symptoms in the GP practice was another factor pushing GPs in certain directions:

*'Especially in the middle of the week, when you know ... the patient can contact you again in two or three days. Then it’s a relaxed situation, and then you can always adjust again and try it first* [without antibiotics].' (B18)

In this context, a notable aspect pertained to reimbursement by statutory health insurance, which offered GPs certain factors that eased their decision-making processes. For example, referring to herbal treatments, while patients *'are stuck with the costs* [...] *it’s easier to hand out a prescription that cost nothing'* (B10).

### Factors complicating the decision making

Under this category, we collected the following sub-themes complicating the decision-making process: diagnostic and prognostic uncertainty; scepticism towards evidence-based recommendations; and discordance within the doctor–patient relationship.

#### Diagnostic and prognostic uncertainty

Situations with higher uncertainty regarding the diagnosis or the prognosis, for example, dealing with pregnant women, older patients, or relapsing UTIs, seemed to make the decision making difficult:

*'But I find geriatric patients from decision making towards antibiotics yes or no, more difficult than, for instance, a younger patient*.' (B20)

*'Pregnant women with UTIs are ticking timebombs* […]*. Of course, I take a look* [...]*. But in the end, I refer them.'* (B11)

#### Scepticism towards evidence-based recommendations

Scepticism and lack of agreement with available evidence and guidelines make GPs’ reasoning more difficult:

*'Medicine does not always work according to guidelines or algorithms*.' (B19)

Accordingly, one GP stated that they did not consider guideline recommendations when deciding whether to prescribe specific antibiotics (B1, supplement). Some participants reported prescribing fewer fluoroquinolones (second-line antibiotics) owing to the Dear Doctor letters they received (B19, supplement). However, they criticised the warning letters for lacking recommendations on what medications to prescribe instead.

*'Exactly, there’s nothing on what to give instead.'* (B7)

#### Discordance within the patient–doctor relationship

At a relational level, deciding about prescribing antibiotics became more complicated when GPs' and patients' prerogatives conflicted:

*'I do try to change my patient’s mind, first. But sometimes,* [...] *if one can find a reasonable justification to prescribe it in spite of everything, then I do it sometimes do it.* [...] *But levofloxacin or ciprofloxacin, I wouldn’t prescribe that right away, if someone wanted that right away*.' (B17)

'[Answering the question: If someone insists on receiving an antibiotic?] *He doesn't get a prescription. I am still in charge of the decision of what the patient takes.'* (B14)

Discordance was fostered by GPs feeling that patients sometimes have a lack of trust or comprehension. Participants reported the doctor–patient relationship being challenged by patients’ demands and by an attitude reported for some patients, namely that they have more trust in subspecialists (that is, urologists or gynaecologists) than in their GP:

*'But if the specialist writes it down, then for many patients it is just* [...] *I am, so to speak, excluded as a GP.* [...] *On the other hand, others trust a GP if you argue well. So, it can be very different*.' (B4)

Decisions were also complicated when GPs felt they were accused of not being able to understand the patient’s experience:

*'*[...] *the standard saying is, you are a man, you have no idea, it does not hurt you, doctor. Yes. This is standard*.' (B16)

### Consultation modalities

What also impacted the decision making was the way participants reported they engaged in the consultation. We collected the following sub-themes: effects of a longer and more in-depth examination; and shared decision making.

#### A more in-depth consultation

A more detailed history, further testing, antimicrobial susceptibility testing, and a referral to a subspecialist were described as potentially valuable to uncover 'something else behind' more complex and uncertain cases, such as when the UTI relapses frequently:

*'*[...] *I start all over again and see, what’s the matter, again anamnesis, urine test, then, like I said, a susceptibility test from the beginning, if it was already the second, third time* [of UTI]*. And yes, if the relapses are frequent, we also recommend a further urological consultation, yes*.' (B10)

Engaging in more in-depth consultations meant having more chances to have the patient on your side in decisions. In such cases, some GPs stated that they could tell patients, *'I'll give you a prescription for an antibiotic,* [...] *wait for another day and then only redeem the prescription if you continue to have complaints*' (B4).

However, GPs criticised the fact that detailed patient consultations are not remunerated sufficiently. One participant explained this, stating that health insurances do not pay to *'seek the conversation'* with the patient, making it difficult to *'go through the points rationally'* and to *'examine them and observe them as to what really is their problem'* (B16). The same GP believed that if one could have this kind of conversation, *'you don’t need a lot of things because you can then decide correctly'* (B16).

#### Shared decision making with patients

GPs reported that they discuss clinical findings and different treatment options in light of the woman’s wishes, sharing the decision of whether to prescribe an antibiotic or not with the patient to *'find a solution together*' (B20):

*'Then you can discuss that with the patient too, whether she needs an antibiotic or whether she’s willing to, or rather wants to treat it without antibiotics and treat it with a lot of fluid and analgesics*.' (B3)

GPs stated that they gave safety-netting advice and used backup antibiotic prescriptions to be filled in only if symptoms did not resolve (B18, supplement). This was perceived as helpful in the decision against immediate antibiotics:

*'You can negotiate with the patient and say I'll give you a prescription. You try this and that first, and if it doesn't get better, redeem it*.' (B21)

In this context, a long-term relationship built on mutual trust seemed helpful. It appeared to promote patient adherence to therapy, even in cases where the prescribed therapy did not match the patient’s expectations:

*'Yes, if you have a patient, you've known forever, if you make it clear to them, watch out, that’s really nonsense, then they usually believe you*.' (B16)

## Discussion

### Summary

In our study, we identified multiple factors perceived by GPs as either facilitating or complicating a straightforward management decision when dealing with women with uUTIs. Thus revealing the complexity behind the GPs’ reasoning for this apparently straightforward condition. Evidence-based recommendations aided GPs in our study in their decision making, as did GPs’ and patients’ prior experiences with antibiotics. However, depending on the experiences, following these might lead to a prescription behaviour differing from guideline recommendations. This equally applies to following perceived or actual patients’ expectations and demands. Furthermore, organisational constraints facilitate the decision making in uUTI management. For instance, time pressure, such as missing health insurance coverage of alternative treatments, makes GPs prescribe antibiotics. Notably, the day of the week that the patient presents with UTI symptoms can lead to either an antibiotic prescription or waiving.

Other factors complicate GPs’ decision-making processes, such as diagnostic and prognostic uncertainty, scepticism towards evidence-based recommendations, and discordance within the patient–doctor relationship. Finally, we found that certain consultation modalities also affect GPs’ prescription behaviour in uUTI management without necessarily pushing them in one direction or making them question their decision. For instance, more in-depth consultation and a shared decision-making process with patients can both aid in making patients accept a wait-and-see approach. However, both take up a consultation time that GPs feel insufficiently paid for.

### Strengths and limitations

We interviewed participants primarily from rural practices surrounding Würzburg, Bavaria, and northern Baden-Württemberg. GPs in other contexts, such as large cities or countries, may hold different perspectives. However, in line with qualitative research principles, we aimed to represent a trustworthy picture of GPs’ views on managing women’s acute uUTIs. To enhance credibility and dependability, we questioned all the participants iteratively to get in-depth data rather than surveying them. Further, the coding process was also iterative, and the analyses entailed multiple steps involving analysts with different backgrounds and an external researcher (investigator triangulation). While qualitative research samples such as ours are not designed to achieve representativeness, the findings can still be transferrable to similar contexts in similar health systems when they share common structures, policies, or characteristics.

However, some limitations need to be mentioned. First, GPs in training were excluded from this study, as we considered them a different population that would require a separate analysis. Further studies are needed to explore the perspectives of GP trainees as they may substantially differ from those of the actual GP workforce.

Second, we cannot rule out that some GPs, during the interviews, tried to give the 'right' answers (social-desirability bias), although we believe that the distortion of answers is limited in our case. This is because the interview-guide questions were designed not to imply a 'correct' answer, and the interviewer was a medical doctoral student, so that the participants were unlikely to feel intimidated. Moreover, the antibiotic stewardship purposes of the investigation were not revealed to the interviewees.

Third, we kept interviews relatively short (mean duration excluding informed consent and any interruption: 15 minutes, range 8–20 minutes) to enhance acceptability. This may have limited our ability to gather in-depth data.

Fourth, at the time of data collection, the University of Würzburg ethics committee did not allow us to store or report any information directly linked to interview data that could potentially lead to identify study participants, such as age or sex; this may have limited our analyses or the interpretation of results.

### Comparison with existing literature

We found that evidence-based recommendations are helpful to GPs in making appropriate prescribing decisions. Even though some scepticism and lack of agreement with recommendations, based on their own or the patient’s personal experiences and beliefs, were reported. For instance, a participant stated to be '*a big fan of fluoroquinolones'*, which is still an overused antibiotic class in German primary care.^36^ However, in line with recommendations of regulatory agencies worldwide,^37,38^ international clinical practice guidelines,^4,39^ as well as guidelines from other countries,^5,40,41^ in Germany fluoroquinolones are not recommended as first-line antibiotics for acute cystitis.^42^ Instead, first-line antibiotics for uUTI include fosfomycin, nitrofurantoin, trimethoprim, pivmecillinam, and nitroxoline.^42^ Similarly, we found that prior experiences and beliefs on first-line drugs, such as fosfomycin, influenced prescribing, with some GPs questioning the utility and efficacy. As discussed elsewhere,^8,20,43,44^ this suggests that more practice-near guideline implementation and dissemination efforts are still required to foster evidence-based practice.

GPs perceived patients’ demands and preferential trust in subspecialists as hampering the doctor–patient relationship, thus complicating the decision making. This finding needs to be interpreted in light of the fact that in Germany, most ambulatory physicians, including GPs and subspecialists, are self-employed with a contract to a statutory health insurance fund. Patients can visit different GPs or subspecialists at any time without restrictions or co-payment. Thus, UTIs can be treated by GPs as well as by subspecialists such as gynaecologists or urologists. As the earnings of ambulatory physicians depend on a mix of fee-for-service and capitation payments in this system, they also compete with each other economically.^45,46^ This could be one of the reasons why some participants declared they tend to fulfil requests for antibiotics with little questioning. However, in line with these findings, previous studies have shown that adherence to patient demands is widespread in antibiotic prescribing decisions in general^47–49^ and specifically related to UTIs in women.^50^ Our study found that lacking consultation time may incline the GPs’ decision towards immediate antibiotics, although alternative treatments, such as anti-inflammatory drugs or herbal formulations, are available^5,42^ and often acceptable or even desirable from the patient’s perspective.^51^ Previous findings from the UK also suggested that limited consultation time may influence GPs to opt for antibiotic treatment without fully exploring alternative uUTI management strategies, despite national guideline recommendations.^1,5,52^ In Germany, patients can access their GP without restriction or charge. However, GPs must keep consultations as short and infrequent as possible to guarantee economic sustainability.^53^ This applies, in particular, to acute diseases with low complication rates, such as a uUTI. In line with this interpretation, previous studies linked the shortage of GP time with miscommunication.^29^ They suggested that enough resources, such as consultation time, are essential to keep prescriptions of antibiotics low.^54^

Our analyses also suggest that milder UTI symptoms, having enough time to discuss treatment options, and sharing prescribing decisions with patients may favour a more complex decision-making process, not leading automatically to immediate antibiotics. Indeed, a recent qualitative study from the UK showed that women are keen to delay antibiotic treatment in favour of a 'natural' alternative in case of milder illness or no upcoming engagements.^50^ A Dutch observational study found that when patients were given sufficient time to discuss treatment options and information about the potential benefits and risks of antibiotic use, they were more likely to consider and agree to non-antibiotic approaches.^55^ Similar findings were reported in other countries, for example, in Germany,^51^ the UK,^52^ and the Republic of Ireland,^28^ and were recently confirmed in a qualitative interview study conducted among 14 Dutch women who had experienced UTI:^56^ women emphasised the importance of being understood by their GP. They expressed a desire for shared decision making in treatment choices.

Our analysis suggests that the GPs' uncertainty about the diagnosis or prognosis made the prescribing decision more difficult. Previous studies linked the perception of uncertainty by GPs to excessive antibiotic prescribing for respiratory tract infections (RTIs).^18,47,57^ In the case of RTIs, the main reason for the uncertainty is the differentiation between viral and bacterial infections. In contrast, no such differentiation is relevant to UTI management, which always has bacterial aetiology.^58^ Our participants' perceptions of uncertainty likely reflects gaps in the evidence regarding the diagnostic approaches towards UTI^59^ and the prognostic or risk factors for complications.^6^

### Implications for research and practice

Our study contributes to a better understanding of GPs’ decision-making processes when treating women with uUTIs by giving a nuanced picture of the factors influencing this process. Our findings provide reference points for both further studies and policy decisions.

The relevance of personal experiences, compliance with patient demands, and some scepticism towards evidence-based recommendations in prescribing decisions suggest the need for further research on guideline adherence, reasons for non-adherence, and guideline implementation for managing acute cystitis in women. To reduce uncertainty in the clinical management of patients, more easy-to-follow evidence-based recommendations to support GPs in their decisions are needed. Our findings, together with previous studies, suggest to practitioners and policymakers that allocating sufficient consultation time for cases of *simple* acute cystitis and supporting shared decision making contributes to the preservation of antibiotics as crucial tools for treating complicated infections.

## Data

The datasets analysed during the current study are available from the corresponding author on reasonable request.
